# Pneumomediastinum after Tooth Extraction

**DOI:** 10.1155/2016/4769180

**Published:** 2016-02-16

**Authors:** Ilhan Ocakcioglu, Serhat Koyuncu, Mustafa Kupeli, Oguzhan Bol

**Affiliations:** ^1^Department of Thoracic Surgery, KBÜ Karabük Training and Research Hospital, 78100 Karabük, Turkey; ^2^Department of Emergency, KBÜ Karabük Training and Research Hospital, 78100 Karabük, Turkey; ^3^Department of Thoracic Surgery, Medical Faculty, Gaziosmanpasa University, 60100 Tokat, Turkey; ^4^Department of Emergency, Kayseri Training and Research Hospital, 38100 Kayseri, Turkey

## Abstract

Pneumomediastinum is defined as the presence of air in mediastinum. Pneumomediastinum can sometimes occur after surgery. Pneumomediastinum seen after dental procedures is rare. We presented the case of subcutaneous emphysema developed in the neck and upper chest after tooth extraction and discussed the possible mechanisms of pneumomediastinum.

## 1. Introduction

Mediastinal emphysema is defined as the presence of air in the mediastinum after blunt, penetrating, or barotrauma following deterioration of integrity of the tracheobronchial system or digestive system. Although it is a life-threatening situation, it generally creates a self-limiting condition and has no dangerous potential. Subcutaneous and mediastinal emphysema are rare complications that can occur after routine tooth extraction. The reason seen after tooth extraction is using the tool that works with compressed air in dental procedures [1, 2]. This case that has a history of tooth extraction a week ago is presented since it is seen rarely.

## 2. Case Report

**Figure 1:**
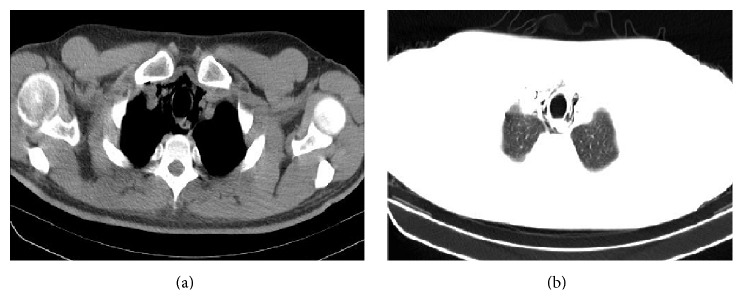
The view of mediastinal emphysema in thorax CT.

**Figure 2:**
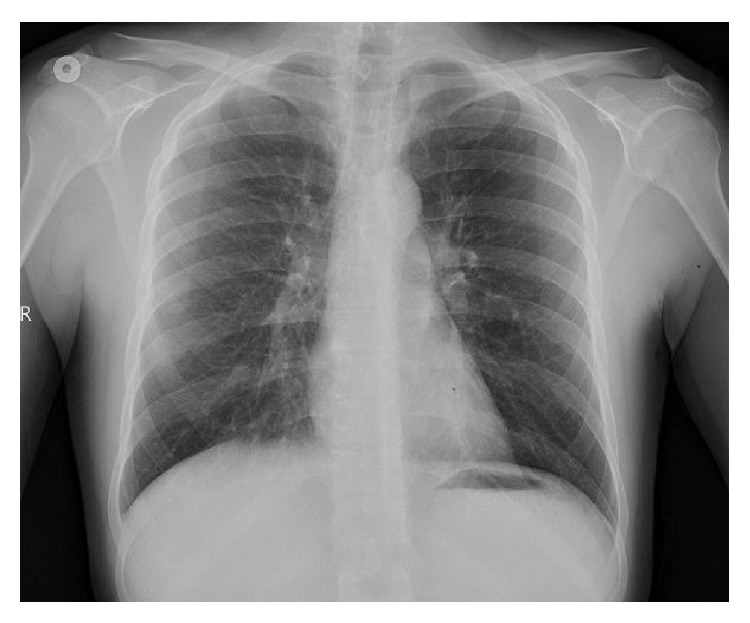
The chest X-ray of patient in the first month.

Twenty-three-year-old male patient with progressive sore throat, shortness of breath, and increased pain on swallowing was admitted to the emergency room. The history of lower right third molar tooth extraction which occurred a week ago was learned. It was learned that the patient did not use mouthwash after tooth extraction. Physical examination showed that heart rate was 77 bpm, blood pressure was 130/80 mmHg, respiratory rate was 20 breaths per minute, body temperature was 36.7°C, and oxygen saturation was 91%. Any open wound could not be found in the oral examination. He had swelling in the neck and chest in inspection. There was subcutaneous crepitus under the skin along the anterior chest wall and neck in palpation. Subcutaneous emphysema and pneumomediastinum starting from the neck through mediastinum were identified (Figure 1). Esophagography was requested for the purpose of assessing the integrity of esophagus. An esophageal rupture that could cause mediastinal emphysema was not observed. He was followed up in service with nasal 5 lt/min 100% O_2_ and broad-spectrum antibiotics. Swelling in the neck and crepitation felt in palpation were markedly reduced. Any additional intervention was not needed. Patient was discharged on the 4th day. In the first month of follow-up, he did not have any complaint and chest X-ray and physical examination findings were found to be normal (Figure 2).

## 3. Discussion

Pneumomediastinum is a condition characterized by the presence of air in mediastinum. It may occur due to iatrogenic, traumatic spontaneous, or infectious lesions. It can develop iatrogenically secondary to head and neck surgery, intubation, mechanical ventilation, esophageal perforation, and dental surgery. Occasionally during dental procedure it may be caused by using high-speed air turbine dental drill [1, 3]. In our case, a history of tooth extraction using compressed air equipment was available. Mediastinal emphysema was first reported in 1900 by Turnbull who emphasized that it was associated with the use of instruments providing compressed air in applications of tooth extraction. Air is assumed to be forced into the subcutaneous, facial, and mediastinal space. Large amounts of air diffuse into the retropharyngeal space. In particular, the 1st, 2nd, and 3rd roots of the molar teeth are linked directly to the sublingual and submandibular area. Air passes from here to the mediastinum and creates mediastinal emphysema [2]. Subcutaneous and mediastinal emphysema can be seen after the severe sneezing [4].

Air seen in mediastinum in radiologic examination is diagnostic. Our patient was diagnosed with thoracic CT. Retrosternal pain and shortness of breath are the characteristic signs of pneumomediastinum. A small amount of air passes into the tissue in the milder form of subcutaneous emphysema. As a result, it can cause mild swelling and crepitus on palpation. If large amount of air passes into the tissue, it can cause pressure on airways or dysphagia.

In literature searching, mediastinal emphysema is seen usually after lower third molar tooth extraction [5]. Also in our patient, mediastinal emphysema was detected after third molar tooth extraction. In more than 90% of cases, mediastinal emphysema occurs within the first 1 hour after the tooth extraction [6]. Apart from that, mediastinal emphysema developing cases one week after intervention have been reported in the literature [7]. In our case, mediastinal emphysema developed and the patient was admitted to hospital one week after tooth extraction.

The treatment of subcutaneous and mediastinal emphysema is usually conservative. Usually 100% O_2_ inhalation is applied. Broad-spectrum antibiotics are used in mediastinal emphysema which develops after tooth extraction, if there is a risk of mediastinitis which is developed by oral flora [8]. Subcutaneous and mediastinal emphysema are usually self-limiting and additional treatment is not needed.

Mediastinal emphysema is a life-threatening condition and can be seen after the intervention related to teeth. It is important and a rare complication. In order to prevent this complication, the use of compressed air should be avoided in dental practice and the history of tooth extraction should be investigated in mediastinal emphysema detected in patients.
